# The evolution of phenotypic plasticity under global change

**DOI:** 10.1038/s41598-017-17554-0

**Published:** 2017-12-08

**Authors:** Emma M. Gibbin, Gloria Massamba N’Siala, Leela J. Chakravarti, Michael D. Jarrold, Piero Calosi

**Affiliations:** 1 Département de Biologie Chimie et Géographie, Université du Québec à Rimouski, 300 Allée des Ursulines, Rimouski, Québec G5L 3A1 Canada; 2 Laboratory for Biological Geochemistry, School of Architecture, Civil and Environmental Engineering, École Polytechnique Fédérale de Lausanne (EPFL), Lausanne, Switzerland; 3 College of Science and Engineering, James Cook University, Townsville, 4811 Queensland Australia; 4 Centre d’Ecologie Fonctionnelle et Evolutive (CEFE- CNRS), Montpellier, Cedex 5 UMR 5175 France

## Abstract

Marine ecosystems are currently in a state of flux, with ocean warming and acidification occurring at unprecedented rates. Phenotypic plasticity underpins acclimatory responses by shifting the mean phenotype in a population, which may buffer the negative effects of global change. However, little is known about how phenotypic plasticity evolves across multiple generations. We tested this by reciprocally-transplanting the polychaete *Ophryotrocha labronica* between control and global change scenarios (ocean warming and acidification in isolation and combined) over five generations. By comparing the reaction norms of four life-history traits across generations, we show that juvenile developmental rate in the combined scenario was the only trait that changed its plastic response across generations when transplanted back to control conditions, and that adaptive plasticity was conserved in most traits, despite significant levels of selection and strong declines in individual fitness in the multi-generational exposure. We suggest the change in level of plasticity in the combined scenario is caused by differential allocation of energy between the mean and the plasticity of the trait along the multigenerational exposure. The ability to maintain within-generational levels of plasticity under global change scenarios has important eco-evolutionary and conservation implications, which are examined under the framework of assisted evolution programs.

## Introduction

Phenotypic plasticity, the capacity of a single genotype to produce a range of phenotypes under different environmental conditions^1^, is commonly perceived as being advantageous because it facilitates the persistence of species in spatially and temporally-heterogeneous environments^2^. However, it can be adaptive, non-adaptive or neutral depending on its relation to the optimal fitness in the new environment^3^. If the plastic response evolves in the same direction as that favoured by directional selection, then it is considered adaptive^3^. In contrast, if the plastic response correlates negatively with the average fitness across environments, then it is considered to be non-adaptive (or maladaptive)^3,4^. Non-adaptive plasticity produces phenotypes that are less fit in the new environment. This typically arises when the costs associated with plasticity outweigh the potential benefits, but it can also occur indirectly, if selection results in an energetic trade-off between traits and plasticity is itself considered as a trait: i.e. a positive change in one trait is compensated by a negative change in another^5–7^. Finally, plasticity is considered neutral if it has no effect on fitness but there is no energetic cost imposed by the new environment. Whether or not plasticity favours adaptive evolution in new environments, is debatable^3^. Historically, plasticity has been viewed as an impediment for adaptive responses because it can prevent the selection of the optimum genotype^8,9^. However, recently this view has been revised because of the time it can buy for adaptive responses to occur in species and because of the potentially important role that non-adaptive plasticity plays in enhancing selective responses to environmental perturbations^4^.

There is renewed interest in understanding the relationship between plasticity, selection and adaptation because of their important role in determining a species capacity for trans-generational acclimatisation and rapid adaptation during periods of fast environmental change, such as those occurring and predicted to occur as a consequence of anthropogenic activities during the 21^st^ century^10–13^. Parental exposure to (future) ocean warming conditions for example, enhanced aerobic scope and growth in fish^14–17^, while exposure to ocean warming and acidification in combination reconciled reductions in the size of coral larvae^18^. Similar, positive parental effects have been documented in copepods^19,20^, oysters^21,22^ and polychaetes^23–25^. Yet, we know little about the persistence of these plastic responses and their potential to promote adaptation to global change scenarios across multiple generations.

Reaction norms provide an excellent means of visualising and evaluating phenotypic plasticity^3,6,26^. If there is sufficient genetic variation to support plastic responses, and the changes in plasticity have a direct effect on fitness, a change in the slope of the reaction norm across generations and environments can indicate the evolution of plasticity, while the direction of the change can indicate whether the response is adaptive, non-adaptive or neutral. Specifically, if the slope of reaction norm changes across generations in accordance with the optimum mean trait selected in the new environment, then we can conclude that plasticity is adaptive. On the other hand, if the reaction norm flattens over time and diverges from this optimum, then we expect a loss of plasticity in the new environment and an increase in genetic canalisation^27^: i.e. the ability to produce the same phenotype regardless of the variability of the genotype or the environment^28^. Reductions in plasticity occur for various reasons. Plasticity can be lost if it is too energetically costly to maintain^9^, or it can be lost through genetic mutations if a selective advantage is gained by knocking out specific pathways^29^.

Multi-generational experiments lasting longer than two generations are scarce, however two recent studies have identified three generations as being the minimum exposure period required for the benefits of parental conditioning to be lost and for negative effects to accumulate^17,30^. The first study exposed the marine stickleback *Gasterosteus aculeatus*, to ocean warming for three generations^17^, while the second exposed the marine polychaete, *Ophryotrocha labronica* La Greca & Bacci, 1962 (Dorvilleidae)^31^ to ocean warming and acidification, in isolation and combination for six generations^30^. Both studies report reductions in hatching event success under warming conditions (72 and 57%, relative to control conditions, respectively), indicative of high levels of selection. Moreover, both studies describe significant changes in key fitness-related life-history traits such as juvenile development rate, average reproductive size and fecundity^17,30^. In particular, Gibbin *et al*.^30^ reported that multiple generations of exposure to both ocean warming and ocean warming in combination with ocean acidification produced faster-growing polychaetes, which reproduced earlier, but at a smaller size, resulting in reduced reproductive output. This could be caused by a simple re-allocation of energetic resources between traits. However it is equally possible that the energy required to fuel the faster growth rates is obtained at the expense of other less obvious mechanisms, such as phenotypic plasticity. We hypothesised that individuals exposed to constant, physiologically-demanding environments for multiple generations would preferentially allocate energy into maintaining fitness, at a cost to the level of plasticity. We had the opportunity to test this theory using the results of a second experiment *run in parallel* to the multi-generational exposure^30^, where we reciprocally-transplanted progeny at generations F3, F4 and F5 between control and global change scenarios: ocean warming and acidification in isolation and combined. The aim of this study was to assess whether phenotypic plasticity can rapidly evolve under global change scenarios and if so, to determine whether the changes were adaptive, non-adaptive or neutral.

## Methods

Adult individuals of the marine polychaete *Ophryotrocha labronica*, were collected in Porto Empedocle harbour (Sicily, Italy: 37°17′N, 13° 31′E) in January 2014 and transferred to the Marine Eco-Evolutionary Physiology (MEEP) laboratory at the Université du Québec à Rimouski (Canada). *Ophryotrocha labronica* is an emerging model for evolutionary studies because of its simple and relatively rapid life cycle and the ease with which it can be cultured^23,24,30,32^. At 27 °C, larval competency takes approximately one week to complete. The juvenile phase lasts an additional week before sexual maturity is reached. At this point, mature adults form breeding pairs and egg masses are laid on a semi-continuous basis, at a rate of approximately one *per* week.

Twelve females and males were randomly selected from the starting population and paired to form the F0 generation^24^. The breeding pairs were kept in six-well plates (Corning, Wiesbaden, Germany), and exposed to a 12 h light: 12 h dark regime. The polychaetes were fed a diet of minced spinach and cleaned daily throughout the experiment to avoid the accumulation of toxins and prevent undesired fermentation. Subsequent generations were always derived from the second egg mass since this mass is largest^24,30^. Upon hatching, 100 offspring were collected and evenly distributed between four scenarios: control (27 °C, pH 8.1), ocean acidification (27 °C, pH 7.6), ocean warming (30 °C, pH 8.1) and their combination (30 °C, pH 7.6), designed to mimic global change predictions for 2100^10^. Conditions were achieved using the temperature and CO_2_-controlled system located in the MEEP laboratory^24^. The polychaetes were exposed to the global change scenarios for six generations (F1-F6). At each generation, 25 hatchlings were collected and transferred to new culture plates, which were kept in the same treatment as their parents. It is important to highlight that not all egg masses hatched at the first attempt. Success rates were high in the control and ocean acidification scenarios (98 and 100%, respectively), reduced in the warming scenario (57%) and lowest in the combined scenario (43%). In the event of a hatching failure, a new breeding pair was created using females from the same brood. A second failure resulted in the creation of a third partnership, but any subsequent failures were categorised as ‘extinction events’, meaning that no further efforts were made to save that lineage (6% of all egg masses lost). The effects of this multi-generational exposure are provided in Gibbin *et al*.^30^.

**Figure 1:**
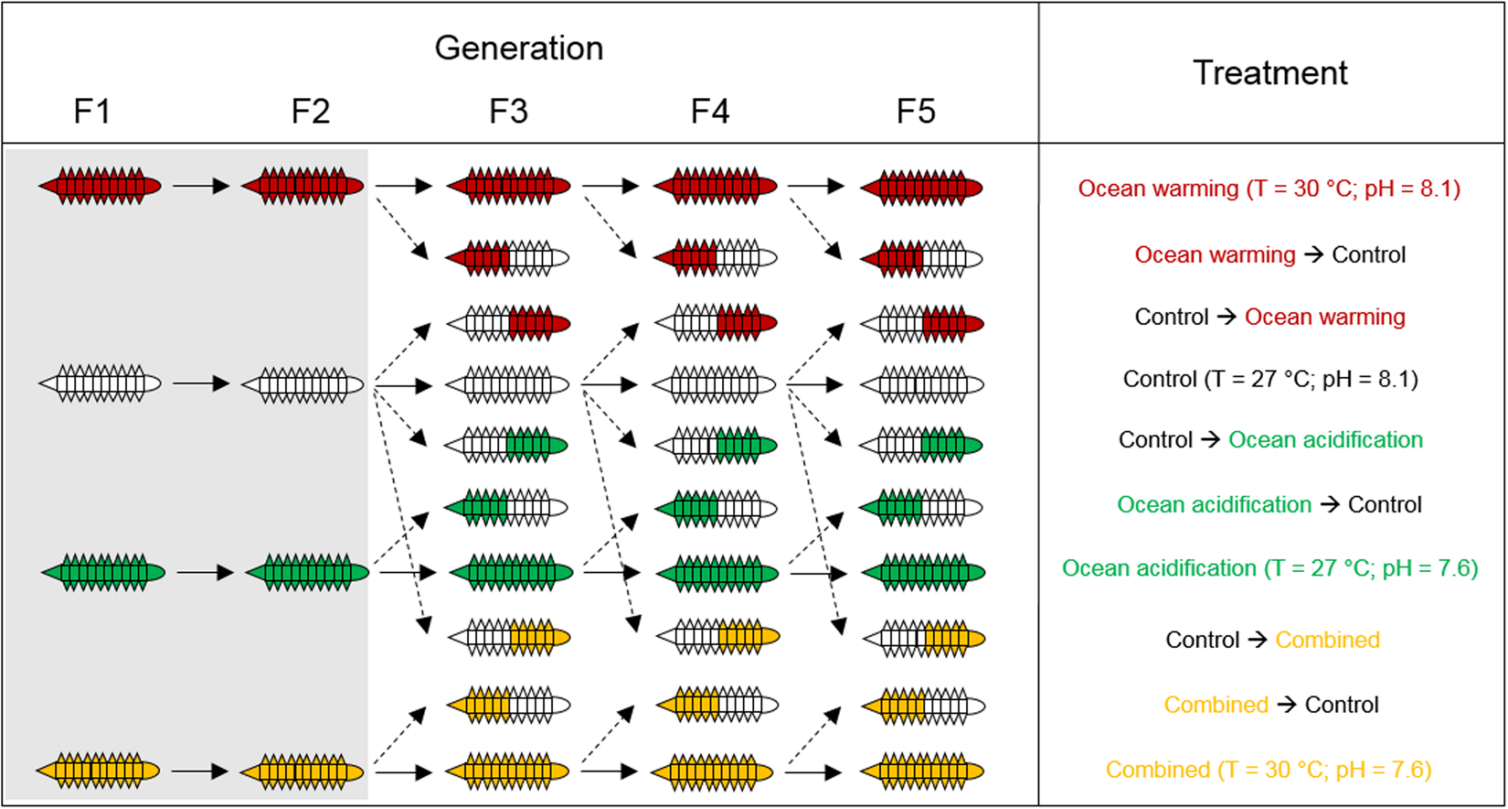
Schematic diagram of the experimental design. Adult individuals of the polychaete worm *Ophryotrocha labronica* (*n* = 12 pairs) were exposed to either control (27 °C, pH 8.1, white), ocean warming (30 °C, pH 8.1, red), ocean acidification (27 °C, pH 7.6, green) or their combination (30 °C pH 7.6, yellow). Reciprocal transplants (dashed arrows) were carried out between experimental and control conditions in F3, F4 and F5. Solid arrows show matching parental and offspring conditions. Polychaetes are colour coded to show the conditions they originated in (left half) and those that they were transplanted to (right half).

We reciprocally-transplanted progeny between control and experimental conditions in generations F3, F4 and F5. The F3 generation was chosen as the starting point for the transplants because it corresponded with the first occurrence of extinction events in the ocean warming and combined scenarios and thus, represented the highest levels of selection observed up until this point^30^. Offspring were obtained as described before, from the second egg mass, but this time, 25 hatchlings were removed from each replicate and transplanted back to control conditions (Fig. 1) and 75 hatchlings were removed from each control replicate and distributed evenly between the different global change scenarios (Fig. 1). The hatchlings were monitored following the protocol below, for a single generation in their new environment and then discarded (Fig. 1).

Four traits were measured at each generation: juvenile developmental rate, survival to sexual maturity, body size at reproduction and fecundity. All measurements required the use of a light microscope (MS5, Leica, St Gallen, Switzerland). Juvenile developmental rate *per* day was expressed as the number of segments bearing chitinous bristles (chaetigers) standardised by time (in days), and was measured exactly one week after the hatchlings were transferred to their new environment. Survival to sexual maturity, defined as the day that oocytes were observed for the first time in females and jaws had visibly developed in males^30^, was expressed as the percentage of individuals that were alive at this point. Coinciding with this, females were paired with males from a different brood that had experienced the same conditions as the female. Whenever a new egg mass was spawned, the number of chaetigers the female had was counted and used to calculate the average body size at reproduction over a reproductive period spanning four spawning events. Fecundity was quantified from the number of eggs present in egg masses 1, 3 and 4. Eggs were counted using ImageJ (National Institutes of Health, Bethesda, MA, USA), from photographs taken on a digital camera (14 MP, Omax, Bucheon, South Korea).

Plasticity was assessed using Univariate Analyses of Variance with “Transplant” (control, ocean warming, ocean acidification or combined) and “Generation” (F3, F4 or F5) as fixed factors. Since the experiment did not employ a full-factorial design, global change drivers and the control were analysed separately. In the event of a significant Transplant × Generation interaction being detected, a *t*-test was performed in each generation to compare the mean of the trait in the new environment (following transplantation) with the mean of the trait after multiple generations. This comparison enabled us to determine whether the within-generation plastic response was adaptive or non-adaptive^3^. A second *t*-test was used to compare the mean value of the trait in the new environment with the mean of the value of the trait in the original (pre-transplantation) environment. This enabled us to determine whether the plastic response was beneficial, non-beneficial or neutral for the trait. If no significant Transplant × Generation interaction was detected then a minimal model approach was employed whereby the term was removed and the model re-run. In the event of a significant Transplant effect being detected the same two *t-*test comparisons were performed only using pooled data (i.e. all generations together). Normality of the data and homogeneity of variances were confirmed prior to all analyses being conducted using the Shapiro-Wilks and Levene’s tests, respectively. If the data did not meet the assumptions of normality or homogeneity, then the significance of residuals was verified. These were never found to be significant (*P* > 0.05). Data analyses were performed using version 13 of the statistical software JMP (SAS Institute, Cary, NC, USA).

## Results

**Figure 2:**
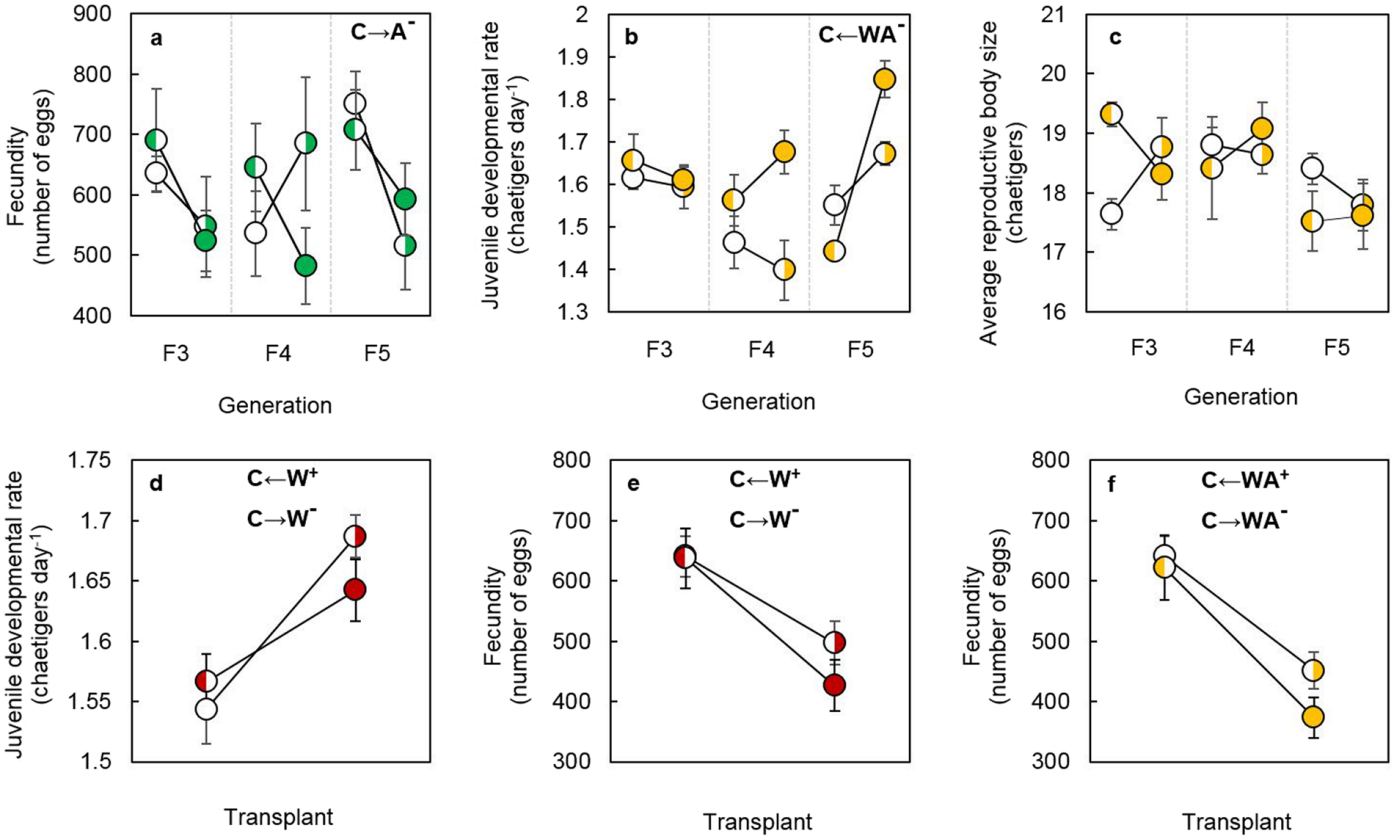
Reaction norms depicting change in phenotypic plasticity of life-history traits. Reaction norms are depicted by solid lines joining the environment that the polychaetes hatched in (solid circles) with the new, post-transplant environment (half-filled circles: left-hand half depicting the environment of origin and right-hand half depicting the new environment). Only traits that had a significant change in their plasticity are shown here: (**a**) fecundity under ocean acidification; (**b**) juvenile developmental rate under combined; (**c**) average reproductive body size under combined; (**d**) juvenile developmental rate under ocean warming; (**e**) fecundity under ocean warming and (**f**) fecundity under combined conditions. The responses of all traits are provided as Supplementary Information (Fig. S1). Colours represent experimental conditions: control (27 °C, pH 8.1, white), ocean warming (30 °C, pH 8.1, red), ocean acidification (27 °C, pH 7.6, green) or their combination (30 °C and pH 7.6, yellow). Values represent the mean change in plasticity ± S.E. The results of *t*-tests, conducted to determine whether plastic responses were beneficial (+) or non-beneficial (−) for the trait are indicated on the figure.

Multigenerational exposure to global change scenarios only modified the level of plasticity of a single trait; juvenile developmental rate, in the combined scenario after transplant to the control (as is indicated by the presence of the significant transplant by generation interaction; Table 1). After two generations (F3, F4) in which no plastic responses were observed following transplant, the rate of juvenile development in the new, control environment significantly decreased compared to the mean measured in the combined scenario. The slope of the reaction norm being three times steeper in F5 compared to F3 (Fig. 2b). Transplantation from ocean warming back to the control significantly modified juvenile developmental rate and fecundity, but the effect of transplant did not change between generations (Table 1). On average, transfer from ocean warming to control resulted in a 5% decrease in juvenile developmental rate (Fig. 2d) and a 49% increase in fecundity (Fig. 2e). There were no changes in trait plasticity for individuals transplanted from ocean acidification to the control (Table 1). However, transplant from combined to the control increased fecundity by 67% (Fig. 2f

**Table 1:** Phenotypic plasticity in life-history traits following multiple generations of exposure to global climate change scenarios.

Origin	Exposure	Source	Trait			
			Juvenile developmental rate	Juvenile survival to sexual maturity	Average reproductive body size	Fecundity
Ocean warming	Control	Transplant	F_1,66_ = 6.113, ***p*** = **0.016**	F_1,66_ = 0.223, *p* = 0.638	F_1,66_ = 0.005, *p* = 0.942	F_1,66_ = 11.189, ***p*** = **0.001**
		Generation	F_2,66_ = 6.262, ***p*** = **0.003**	F_2,66_ = 0.948, *p* = 0.393	F_2,66_ = 0.206, *p* = 0.814	F_2,66_ = 4.433, ***p*** = **0.016**
		Transplant × Generation	—	—	—	—
Ocean acidification		Transplant	F_1,67_ = 2.841, *p* = 0.097	F_1,67_ = 0.040, *p* = 0.842	F_1,67_ = 0.129, *p* = 0.721	F_1,67_ = 2.608, *p* = 0.111
		Generation	F_2,67_ = 26.495, ***p*** = <**0.001**	F_2,67_ = 11.438, ***p*** = <**0.001**	F_2,67_ = 1.431, *p* = 0.246	F_2,67_ = 0.249, *p* = 0.780
		Transplant × Generation	—	—	—	—
Ocean warming and acidification combined		Transplant	F_1,48_ = 14.142, ***p*** = <**0.001**	F_1,50_ = 0.175, *p* = 0.677	F_1,50_ = 0.312, *p* = 0.597	F_1,50_ = 15.782, ***p*** = <**0.001**
		Generation	F_2,48_ = 0.112, ***p*** = **0.894**	F_2,50_ = 6.222, ***p*** = **0.004**	F_2,50_ = 3.458, ***p*** = **0.039**	F_2,50_ = 1.770, *p* = 0.181
		Transplant × Generation	F_2,48_ = 10.019, ***p*** = <**0.001**	—	—	—

F-ratio’s (F) with degrees of freedom and probability levels (*p*) are provided and significant effects (*P* < 0.05) are highlighted in bold. Hyphens (−) denotes terms that were not found to be significant, and therefore removed from the model.

). In all of these cases, the mean trait in the new environment (control) was statistically similar to the mean of the trait measured after multiple generations in the control scenario.

Multigenerational exposure to the control modified the plasticity of two traits: fecundity after transplant into ocean acidification and reproductive body size upon transfer to the combined scenario (Table 2). Both interactions were driven by changes in direction of the reaction norm slope in a single generation: F4 for fecundity and F3 for body size, respectively (Fig. 2a,c). Transplant from control to the ocean warming scenario resulted in a 9% increase in juvenile developmental rate (Fig. 2d) but reduced fecundity by 22% (Fig. 2e), whereas transplant from control to the combined scenario decreased fecundity by 30% (Fig. 2f). However, the level of plasticity in these traits did not change across generations (Table 2). No changes in plasticity were detected in individuals transplanted from the control to ocean acidification (Table 2

**Table 2:** Phenotypic plasticity in life-history traits following multiple generations of exposure to control conditions.

Origin	Exposure	Source	Trait			
			Juvenile developmental rate	Juvenile survival to sexual maturity	Average reproductive body size	Fecundity
Control	Ocean warming	Transplant	F_1,68_ = 19.842, ***p*** = <**0.001**	F_1,68_ = 1.232, *p* = 0.271	F_1,68_ = 0.000, *p* = 0.995	F_1,66_ = 9.044, ***p*** = **0.004**
		Generation	F_2,68_ = 3.408, ***p*** = **0.039**	F_2,68_ = 18.272, ***p*** = <**0.001**	F_2,68_ = 2.238, *p* = 0.114	F_2,66_ = 3.064, *p* = 0.053
		Transplant × Generation	—	—	—	—
	Ocean acidification	Transplant	F_1,67_ = 0.986, *p* = 0.324	F_1,67_ = 2.403, *p* = 0.126	F_1,67_ = 1.673, *p* = 0.200	F_1,65_ = 0.922, *p* = 0.341
		Generation	F_2,67_ = 7.729, ***p*** = **0.001**	F_2,67_ = 16.545, ***p*** = <**0.001**	F_2,67_ = 3.022, *p* = 0.055	F_2,65_ = 0.160, *p* = 0.853
		Transplant × Generation	—	—	—	F_2,65_ = 3.481, ***p*** = **0.037**
	Ocean warming and acidification combined	Transplant	F_1,68_ = 0.075, *p* = 0.785	F_1,68_ = 1.324, *p* = 0.254	F_1,66_ = 0.152, *p* = 0.698	**F**_**1,68**_ = **20.291**, ***p*** = <**0.001**
		Generation	F_2,68_ = 8.223, ***p*** = **0.001**	F_2,68_ = 17.092, ***p*** = <**0.001**	F_2,66_ = 1.820, *p* = 0.170	F_2,68_ = 6.712, ***p*** = **0.002**
		Transplant × Generation	—	—	F_2,66_ = 3.337, ***p*** = **0.042**	—

F-ratio’s (F) with degrees of freedom and probability levels (*p*) are provided and significant effects (*P* < 0.05) are highlighted in bold. Hyphens (−) denotes terms that were not found to be significant, and therefore removed from the model.

).

## Discussion

In this study, we evaluate how phenotypic plasticity evolves across multiple generations in conditions designed to simulate three global change scenarios: ocean warming and ocean acidification, in isolation and combined. Significant changes in the level of plasticity across generations, underlined by the presence of significant transplant by generation interactions, were surprisingly few. Only juvenile developmental rate under the combined scenario modified the transplant effect across generations, pointing to the fact that this trait evolves towards an increase in plasticity. The response of transplant from the combined to the control scenario was steeper in F5, showing a higher level of plasticity compared to that observed in F3 and F4, in which no plasticity was observed. The direction of the plastic change observed in F5 was non-beneficial for the trait, i.e. it reduced the rate of juvenile development in individuals that were transplanted back to control conditions. There was no significant difference between the trait mean of individuals in the new environment following transplant and the trait mean of individuals that had been exposed to combined conditions for five generations, suggesting that the plastic response, which permits transplanted individuals to reach the expected fitness in the new environment, was adaptive^3^. Interestingly, there was no change in the level of plasticity across generations in juvenile developmental rate in the ocean warming scenario, yet adaptive plasticity was maintained (Fig. 2).

The change in the level of plasticity in the combined scenario and not in the ocean warming scenario may be caused by a differential allocation of energy between optimising the mean and maintaining the plasticity of the trait along the multigenerational exposure. Five generations of exposure to ocean warming conditions did result in increase in the mean juvenile developmental rate, but not in the combined scenario^30^. The selection of faster-growing juveniles under ocean warming was accompanied by a reduction in body size at reproduction and a decrease in fecundity^30^. The presence of such trade-offs may explain why plasticity was unable to evolve under ocean warming but fostered in combined conditions. Increased developmental rates are costly, and require compensation in the form of reduced energy allocation to other energetically costly traits and/or functions^33^: e.g. reproduction^20^. Thus, for plasticity in juvenile developmental rate to evolve, the fitness advantage gained from the improved ability to adjust the peak of the trait mean, for example when coping with fluctuating resource availability^34,35^, must be more advantageous than the benefits provided by reaching a larger size at sexual maturity^36^, which in this species is normally linked to higher fecundity^37^.

The maintenance of adaptive plasticity in all of the life-history traits investigated (Table 3) suggests that phenotypic plasticity is not a significant energetic cost to this species^8,38^ at least not over the timeframe involved in this experiment. The conservation of plasticity has clear fitness benefits for individuals exposed to highly variable environmental conditions, like *O. labronica* ^39^ that frequently have to counterbalance the energetic demands imposed by their physiological functions with the challenges levied by a fluctuating environment.

**Table 3:** Summary of the plastic responses observed in life-history traits following transplantation from global change scenarios (ocean warming, W, ocean acidification, A, and their combination, WA) to control conditions (C).

Trait	Transplant	Generation		
		F3	F4	F5
Juvenile developmental rate	W-C	non-beneficial/adaptive		
	WA-C*	neutral/adaptive	neutral/adaptive	non-beneficial/adaptive
	C-W	beneficial/adaptive		
Average reproductive body size	C-WA	neutral/adaptive	neutral/adaptive	neutral/adaptive
Fecundity	W-C	beneficial/adaptive		
	WA-C	beneficial/adaptive		
	C-W	non-beneficial/adaptive		
	C-A*	neutral/adaptive	neutral/adaptive	non-beneficial/adaptive
	C-WA	non-beneficial/adaptive		

Plasticity was considered beneficial when the mean value of the trait in the new environment was higher than the mean of the value of the trait in the original environment; non-beneficial when the mean value of the trait in the new environment was lower than the mean of the value of the trait in the original environment and neutral when the two means were statistically comparable. Plasticity was considered adaptive when the mean value of the trait in the new environment was similar to the mean value of the trait after multiple generations of exposure in the new environment and non-adaptive when it differed (see Fig. 2). If a Transplant × Generation interaction was present (*) the nature of plasticity is provided for each generation. If a Transplant effect was detected the nature of plasticity is provided over all generations. Only scenarios and/or traits displaying significant Transplant × Generation or Transplant effects are shown and statistical output is provided as Supplementary Information (Tables S4, S5).

If we consider the change in plasticity detected in the control line, significant changes in reaction norms across generations were observed for fecundity in the ocean acidification scenario and reproductive size in the combined scenario, but these were not accompanied by a change in the mean of other traits along the multigenerational exposure to control conditions (Table 2). We thus cannot completely discard the idea that other mechanisms contributed in determining these patterns, such as genetic drift or the random sampling of different genotypes across generations. The limited number of lineages used in our study (*n* = 12) could have favoured the contribution of random mechanisms over selective ones. This is particularly true for the ocean warming and combined scenarios, where we observed high levels of early-stage mortality (43% of egg masses lost and 33% loss of lineages lost in F5 under ocean warming; 57% of hatchling mortality and 24 and 25% loss of lineages in F3 and F5, respectively, under combined conditions^30^). All considering, disentangling the relative contribution of these mechanisms, and further investigating the role of energy allocation and trait trade-offs in shaping the evolution of phenotypic plasticity under global change, should be deemed a priority in the investigation of the eco-evolutionary implications of global change on biological systems.

Irrespective of the mechanisms involved, the maintenance of adaptive plasticity over multiple generations under global change scenarios has important implications for conservation purposes. *Assisted Evolution* ^40^, for example, has been championed as one potential means of conserving Earth’s biodiversity in the face of the ongoing global change^24,40–42^. Assisted evolution programs have already been successfully implemented in aquaculture^43,44^, and are gaining momentum in coral reef restoration initiatives^11,40,42^. Three phases exist in such conservation strategies: (i) the induction of heritable changes in tolerance in the parental generation; (ii) the selective breeding of individuals that bear favourable traits and (iii) the transplant of tolerant individuals back to their natural environment. The success of assisted evolution programs relies on striking a balance between enhancing the trait mean(s) of desired trait(s) (e.g. thermal tolerance) while maintaining the ability to respond to other environmental fluctuations that occur within a single generation^40,42^. The model species that we used here, *O. labronica*, was able to retain high levels of adaptive within-generational plasticity, despite being exposed to highly selective, constant global change scenarios over multiple generations. Our findings therefore, lend support to the feasibility of conservation approaches that employ an evolutionary perspective^40,45,46^.

## Electronic supplementary material

Supplementary Information
