# Supplementary material for: The evolution of phenotypic plasticity under global change
Source: Sci Rep. 2017 Dec 8;7:17253. doi: 10.1038/s41598-017-17554-0 (PMC5722875; doi:10.1038/s41598-017-17554-0)
Supplement: Supplementary file 1 — Supplementary Information [file 41598_2017_17554_MOESM1_ESM.pdf]

# Supplementary Information

# The evolution of phenotypic plasticity under global change

Emma M. Gibbin[1,2*], Gloria Massamba N'Siala[1,4], Leela J. Chakravarti[1,3], Michael D. Jarrold[1,3], Piero Calosi[1]

**Figure S1. Reaction norms depicting change in phenotypic plasticity of all life-history traits.** Reaction norms are depicted by solid lines joining the environment that the polychaete hatched in (solid circles) with the new, post-transplantation environment (half-filled circles: left-hand half depicting the environment of origin and right-hand half depicting the new environment). Colours represent experimental conditions: control (27 °C, pH 8.1, white), ocean warming (30 °C, pH 8.1, red), ocean acidification (27 °C, pH 7.6, green) or their combination (27 °C, pH 7.6, yellow). Values represent mean ± SE.

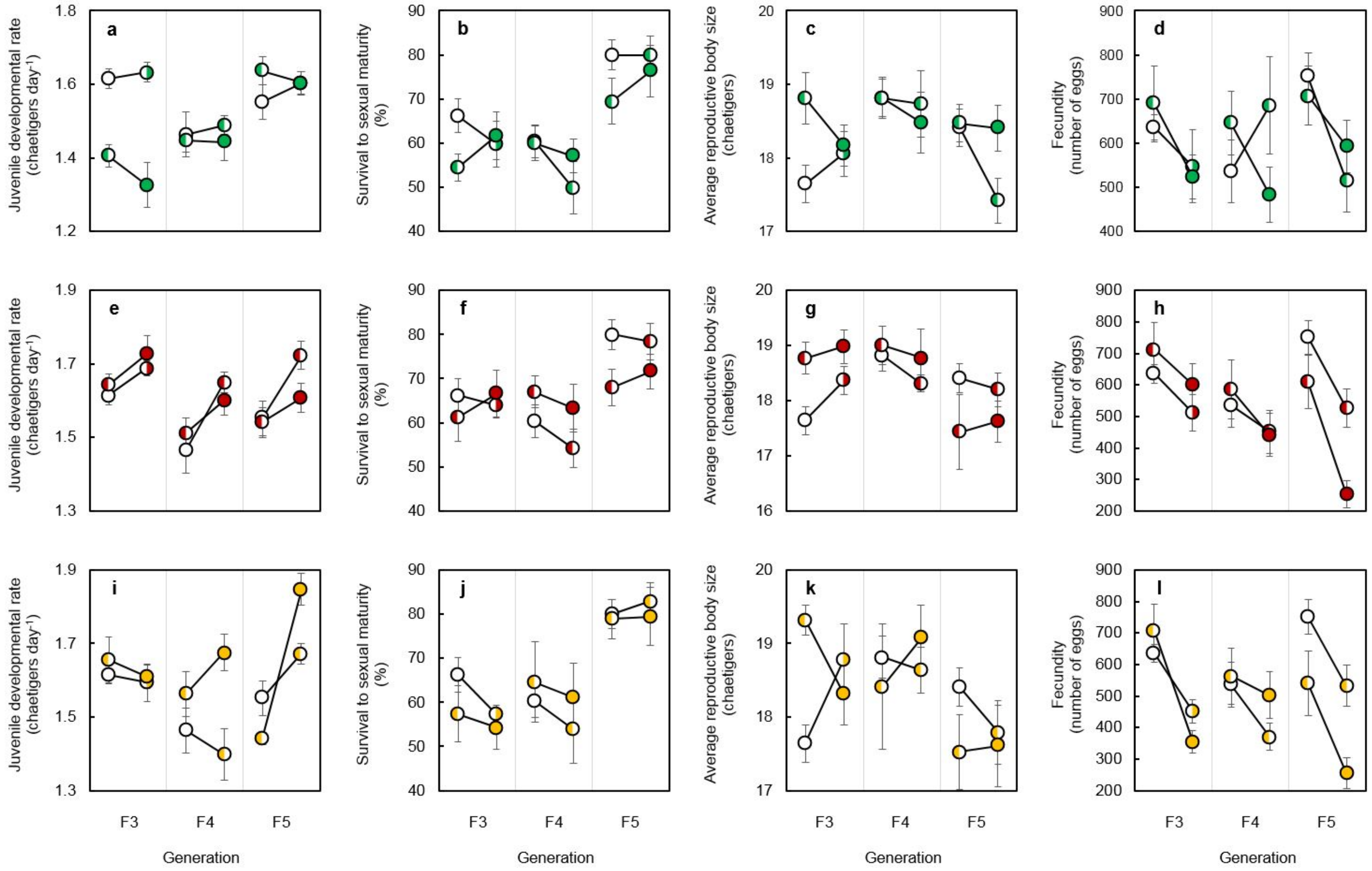

**Table S1. Life-history measurements after multigenerational exposure and reciprocal transplantation between control and ocean warming.** *Ophryotrocha labronica* individuals (N=12) were exposed to control (C; 27 °C, pH 8.1) and ocean warming (W; 30 °C, pH 8.1) for six generations (F1-F6). Transplantations were carried out in generations F3, F4 and F5 by transplanting individuals from multi-generational exposure conditions (either C-C or W-W) to either control or ocean warming (C-W or W-C). These individuals were retained in the new environment for a single generation. Four life-history traits were measured: juvenile developmental rate, survival to sexual maturity, average reproductive body size and fecundity. Values represent mean ± SE and number of replicates are provided in parentheses.

| Life history trait | F3 | | | | F4 | | | | F5 | | | |
|---|---|---|---|---|---|---|---|---|---|---|---|---|
| | C-C | C-W | W-C | W-W | C-C | C-W | W-C | W-W | C-C | C-W | W-C | W-W |
| Juvenile developmental rate (chaetigers $d^{-1}$) | 1.62<br>*0.01*<br>(12) | 1.69<br>*0.01*<br>(12) | 1.64<br>*0.01*<br>(12) | 1.73<br>*0.01*<br>(11) | 1.46<br>*0.02*<br>(12) | 1.65<br>*0.01*<br>(12) | 1.51<br>*0.01*<br>(11) | 1.60<br>*0.01*<br>(12) | 1.55<br>*0.01*<br>(12) | 1.72<br>*0.01*<br>(12) | 1.54<br>*0.01*<br>(12) | 1.61<br>*0.01*<br>(12) |
| Juvenile survival to sexual maturity (%) | 66.21<br>*1.12*<br>(12) | 63.99<br>*0.79*<br>(12) | 61.09<br>*1.54*<br>(12) | 66.55<br>*1.64*<br>(11) | 66.21<br>*1.08*<br>(12) | 54.18<br>*1.23*<br>(12) | 67.00<br>*1.09*<br>(11) | 63.27<br>*1.55*<br>(12) | 80.00<br>*0.97*<br>(12) | 78.40<br>*1.18*<br>(12) | 68.00<br>*1.21*<br>(12) | 71.60<br>*1.11*<br>(12) |
| Average reproductive body size (chaetigers) | 17.64<br>*0.07*<br>(12) | 18.36<br>*0.07*<br>(12) | 18.77<br>*0.08*<br>(12) | 18.98<br>*0.09*<br>(11) | 18.82<br>*0.08*<br>(12) | 18.31<br>*0.05*<br>(12) | 19.00<br>*0.11*<br>(11) | 18.78<br>*0.15*<br>(12) | 18.41<br>*0.08*<br>(12) | 18.20<br>*0.09*<br>(12) | 17.43<br>*0.20*<br>(12) | 17.63<br>*0.11*<br>(12) |
| Fecundity (number of eggs) | 635.25<br>*8.38*<br>(12) | 511.58<br>*16.93*<br>(12) | 712.33<br>*24.53*<br>(12) | 599.73<br>*20.55*<br>(11) | 535.75<br>*20.40*<br>(12) | 451.42<br>*19.88*<br>(12) | 585.64<br>*27.95*<br>(11) | 440.83<br>*19.91*<br>(12) | 750.76<br>*15.68*<br>(12) | 526.47<br>*17.89*<br>(12) | 609.39<br>*24.03*<br>(12) | 253.25<br>*12.57*<br>(12) |

**Table S2. Life-history measurements after multigenerational exposure and reciprocal transplantation between control and ocean acidification conditions.** *Ophryotrocha labronica* individuals (N=12) were exposed to control (C; 27 °C, pH 8.1) and ocean acidification; (A; 27 °C, pH 7.6) for six generations (F1-F6). Transplantations were carried out in generations F3, F4 and F5 by transplanting individuals from multi-generational exposure conditions (either C-C or A-A) to either control or ocean acidification (C-A or A-C). These individuals were retained in the new environment for a single generation. Four life-history traits were measured: juvenile developmental rate, survival to sexual maturity, average reproductive body size and fecundity. Values represent mean ± SE and number of replicates are provided in parentheses.

| **Life history trait** | **F3** | | | | **F4** | | | | **F5** | | | |
|---|---|---|---|---|---|---|---|---|---|---|---|---|
| | **C-C** | **C-A** | **A-C** | **A-A** | **C-C** | **C-A** | **A-C** | **A-A** | **C-C** | **C-A** | **A-C** | **A-A** |
| Juvenile developmental rate (chaetigers $d^{-1}$) | 1.62<br>0.01<br>(12) | 1.63<br>*0.01*<br>(11) | 1.40<br>*0.01*<br>(12) | 1.33<br>*0.02*<br>(11) | 1.46<br>*0.02*<br>(12) | 1.49<br>*0.01*<br>(12) | 1.45<br>0.01<br>(12) | 1.44<br>*0.01*<br>(12) | 1.55<br>*0.01*<br>(12) | 1.60<br>*0.01*<br>(12) | 1.64<br>*0.01*<br>(11) | 1.60<br>*0.01*<br>(12) |
| Juvenile survival to sexual maturity (%) | 66.21<br>*1.12*<br>(12) | 59.81<br>*1.56*<br>(11) | 54.42<br>*0.87*<br>(12) | 61.75<br>*1.63*<br>(11) | 66.21<br>*1.08*<br>(12) | 49.82<br>*1.71*<br>(12) | 60.00<br>*1.12*<br>(12) | 57.09<br>*1.11*<br>(12) | 80.00<br>*0.97*<br>(12) | 80.00<br>*1.25*<br>(12) | 69.45<br>*1.56*<br>(11) | 76.36<br>*1.68*<br>(12) |
| Average reproductive body size (chaetigers) | 17.64<br>*0.07*<br>(12) | 18.05<br>*0.09*<br>(11) | 18.81<br>*0.10*<br>(12) | 18.17<br>*0.08*<br>(11) | 18.82<br>*0.08*<br>(12) | 18.73<br>*0.13*<br>(12) | 18.81<br>*0.07*<br>(12) | 18.47<br>*0.12*<br>(12) | 18.41<br>*0.08*<br>(12) | 17.42<br>*0.09*<br>(12) | 18.47<br>*0.08*<br>(11) | 18.40<br>*0.09*<br>(12) |
| Fecundity (number of eggs) | 635.25<br>*8.38*<br>(12) | 547.00<br>*25.07*<br>(11) | 689.50<br>*24.76*<br>(12) | 523.45<br>*15.25*<br>(11) | 535.75<br>*20.40*<br>(12) | 685.07<br>*31.85*<br>(12) | 645.21<br>*21.09*<br>(12) | 482.50<br>*18.22*<br>(12) | 750.76<br>*15.68*<br>(12) | 515.42<br>*20.89*<br>(12) | 707.27<br>*20.00*<br>(11) | 592.67<br>*17.27*<br>(12) |

**Table S3. Life-history measurements following multigenerational exposure and reciprocal transplantation between control and combined conditions.** *Ophryotrocha labronica* individuals (N=12) were exposed to control (C; 27 °C, pH 8.1) and combined (WA; 30 °C, pH 7.6) for six generations (F1-F6). Transplantations were carried out in generations F3, F4 and F5 by transplanting individuals from multi-generational exposure conditions (either C-C or WA-WA) to either control or combined (C-WA or WA-C). These individuals were retained in the new environment for a single generation. Four life-history traits were measured: juvenile developmental rate, survival to sexual maturity, average reproductive body size and fecundity. Values represent mean ± SE and number of replicates are provided in parentheses.

| **Life history trait** | **F3** | | | | **F4** | | | | **F5** | | | |
|---|---|---|---|---|---|---|---|---|---|---|---|---|
| | **C-C** | **C-WA** | **WA-C** | **WA-WA** | **C-C** | **C-WA** | **WA-C** | **WA-WA** | **C-C** | **C-WA** | **WA-C** | **WA-WA** |
| Juvenile developmental rate (chaetigers $d^{-1}$) | 1.62<br>0.01<br>(12) | 1.59<br>*0.01*<br>(12) | 1.66<br>*0.02*<br>(12) | 1.61<br>*0.01*<br>(12) | 1.46<br>*0.02*<br>(12) | 1.40<br>0.02<br>(12) | 1.56<br>0.02<br>(8) | 1.68<br>*0.02*<br>(8) | 1.55<br>*0.01*<br>(12) | 1.67<br>*0.01*<br>(12) | 1.44<br>*0.01*<br>(7) | 1.85<br>*0.02*<br>(7) |
| Juvenile survival to sexual maturity (%) | 66.21<br>*1.12*<br>(12) | 57.39<br>*0.24*<br>(12) | 57.39<br>*1.84*<br>(12) | 54.32<br>*1.46*<br>(12) | 66.21<br>*1.08*<br>(12) | 53.82<br>*2.22*<br>(12) | 64.61<br>*3.22*<br>(8) | 61.14<br>*2.71*<br>(8) | 80.00<br>*0.97*<br>(12) | 82.91<br>*1.19*<br>(12) | 78.86<br>*1.67*<br>(7) | 79.43<br>*2.50*<br>(7) |
| Average reproductive body size (chaetigers) | 17.64<br>*0.07*<br>(12) | 18.77<br>*0.14*<br>(12) | 19.32<br>*0.06*<br>(12) | 18.32<br>*0.12*<br>(12) | 18.82<br>*0.08*<br>(12) | 18.64<br>*0.09*<br>(12) | 18.42<br>*0.30*<br>(8) | 19.07<br>*0.16*<br>(8) | 18.41<br>*0.08*<br>(12) | 17.79<br>*0.12*<br>(12) | 17.52<br>*0.19*<br>(7) | 17.61<br>*0.21*<br>(7) |
| Fecundity (number of eggs) | 635.25<br>*8.38*<br>(12) | 450.58<br>*10.94*<br>(12) | 707.42<br>*24.56*<br>(12) | 355.33<br>*10.41*<br>(12) | 535.75<br>*20.40*<br>(12) | 370.33<br>*12.46*<br>(12) | 563.25<br>*31.57*<br>(8) | 502.13<br>*26.30*<br>(8) | 750.76<br>*15.68*<br>(12) | 532.58<br>*18.81*<br>(12) | 539.95<br>*38.99*<br>(7) | 255.43<br>*18.32*<br>(7) |

**Table S4. Statistical output from targeted *t*-tests used to determine whether plastic responses were beneficial, non-beneficial or neutral for life-history traits.** *T*-tests were used to compare the mean value of the trait in the new environment with the mean of the value of the trait in the original (pre-transplantation) environment. This comparison enabled us to determine whether the plastic response was beneficial or non-beneficial for the trait (see Fig. 2 and Table 3). Plasticity was considered beneficial when the mean value of the trait in the new environment was higher than the mean of the value of the trait in the original environment; non-beneficial when the mean value of the trait in the new environment was lower than the mean of the value of the trait in the original environment and neutral when the two means were statistically similar. Only traits where a significant Transplant × Generation interaction or a significant Transplant effect was detected were analysed. In the case of a significant Transplant × Generation interaction the nature of plasticity is provided for each generation. If a Transplant effect was detected the nature of plasticity is provided across all generations. T-values, with degrees of freedom and probability levels (*p*) are provided and significant effects ($P < 0.05$) are highlighted in bold.

| Trait | Variable | | Generation | | |
|---|---|---|---|---|---|
| | Group 1 | Group 2 | F3 | F4 | F5 |
| Juvenile developmental rate | W-W | W-C | T (68) = -2.195, ***p* = 0.032** | | |
| | WA-WA | WA-C | T (22) = 0.664, *p* = 0.513 | T (14) = -1.434, *p* = 0.173 | T (12) = -8.836, ***p* = < 0.001** |
| | C-C | C-W | T (70) = -4.309, ***p* = < 0.001** | | |
| Average reproductive body size | C-C | C-WA | T (22) = -2.031, *p* = 0.054 | T (22) = 0.418, *p* = 0.680 | T (22) = 1.227, *p* = 0.233 |
| Fecundity | W-W | W-C | T (68) = 3.251, ***p* = 0.002** | | |
| | WA-WA | WA-C | T (52) = 3.915, ***p* = < 0.001** | | |
| | C-C | C-W | T (70) = 2.923, ***p* = 0.005** | | |
| | C-C | C-A | T (21) = -1.039, *p* = 0.311 | T (22) = 1.140, *p* = 0.267 | T (22) = -2.600, ***p* = 0.016** |
| | C-C | C-WA | T (70) = 4.177, ***p* = < 0.001** | | |

**Table S5. Statistical output from targeted *t*-tests used to determine whether plastic responses were adaptive or non-adaptive for life-history traits.** In the event of a significant Transplant × Generation interaction being detected, a t-test was performed in each generation to compare the mean of the trait in the new environment (following transplantation) with the mean of the trait after multiple generations. This comparison enabled us to determine whether the within-generation plastic response was adaptive or non-adaptive. Plasticity was considered adaptive when the mean value of the trait in the new environment was comparable to the mean value of the trait after multiple generations of exposure in the new environment and non-adaptive when it differed. Only traits where a significant Transplant × Generation interaction or a significant Transplant effect was detected were analysed. In the case of a significant Transplant × Generation interaction the nature of plasticity is provided for each generation. If a Transplant effect was detected the nature of plasticity is provided across all generations. T-values, with degrees of freedom and probability levels (*p*) are provided and significant effects ($P < 0.05$) are highlighted in bold.

| Trait | Variable | | Generation | | |
|---|---|---|---|---|---|
| | Group 1 | Group 2 | F3 | F4 | F5 |
| Juvenile developmental rate | W-C | C-C | T (69) = 0.630, *p* = 0.531 | | |
| | WA-C | C-C | T (22) = -0.610, *p* = 0.548 | T (18) = -1.100, *p* = 0.286 | T (17) = 1.734, *p* = 0.101 |
| | C-W | W-W | T (69) = 1.431, *p* = 0.157 | | |
| Average reproductive body size | C-WA | WA-WA | T (22) = 0.690, *p* = 0.498 | T (18) = -0.823, *p* = 0.421 | T (17) = 0.262, *p* = 0.796 |
| Fecundity | W-C | C-C | T (69) = 0.057, *p* = 0.955 | | |
| | WA-C | C-C | T (61) = 0.319, *p* = 0.751 | | |
| | C-W | W-W | T (69) = 1.273, *p* = 0.207 | | |
| | C-A | A-A | T (20) = 0.141, *p* = 0.811 | T (22) = 0.204, *p* = 0.125 | T (22) = 0.823, *p* = 0.420 |
| | C-WA | WA-WA | T (61) = 1.711, *p* = 0.092 | | |
